# Management of uterine adenomyosis: current trends and uterine artery embolization as a potential alternative to hysterectomy

**DOI:** 10.1186/s13244-019-0732-8

**Published:** 2019-04-27

**Authors:** Riham Dessouky, Sherif A. Gamil, Mohamad Gamal Nada, Rola Mousa, Yasmine Libda

**Affiliations:** 1 Radiology Department, Faculty of Medicine, Zagazig University, Koliat Al Tob Street, Zagazig, 44519 Egypt; 2 Radiology Department, Al-Ahrar Teaching Hospital, Zagazig, Egypt

**Keywords:** Adenomyosis, Uterine artery embolization, Hysterectomy

## Abstract

Adenomyosis is a challenging clinical condition that is commonly being diagnosed in women of reproductive age. To date, many aspects of the disease have not been fully understood, making management increasingly difficult. Over time, minimally invasive diagnostic and treatment methods have developed as more women desire uterine preservation for future fertility or to avoid major surgery. Several uterine-sparing treatment options are now available, including medication, hysteroscopic resection or ablation, conservative surgical methods, and high-intensity focused ultrasound each with its own risks and benefits. Uterine artery embolization is an established treatment option for uterine fibroids and has recently gained ground as a safe and cost-effective method for treatment of uterine adenomyosis with promising results. In this review, we discuss current trends in the management of uterine adenomyosis with a special focus on uterine artery embolization as an alternative to hysterectomy.

## Key points

- Uterine artery embolization (UAE) seems to be the most promising uterine-sparing, minimally invasive treatment option for adenomyosis.
- Results of ongoing randomized controlled trial (QUESTA) will soon show whether UAE can be validated as a treatment option for adenomyosis.
- Ability to preserve fertility will be one of the main factors determining whether UAE can replace hysterectomy in treatment of adenomyosis, but further randomized controlled trials are needed.

## Introduction

Adenomyosis is defined by the abnormal location of endometrial tissue within the myometrium associated with hypertrophy or hyperplasia of the myometrial stroma [1, 2]. Although pathogenesis and etiology of adenomyosis remain unknown, two main theories have been proposed: invagination of the endometrial basal layer and metaplasia of embryonic stem cells [3]. Prevalence of adenomyosis varies widely from 5 to 70% [4–7] with recent studies showing about 20% prevalence [8–10] among which the majority were premenopausal. Despite the absence of specific (pathognomonic) diagnostic features for uterine adenomyosis, typical symptoms include menorrhagia, chronic pelvic pain, and dysmenorrhea [11]. These symptoms are commonly encountered in other gynecological disorders including leiomyomas and endometriosis, often confounding the clinical diagnosis [12].

For more than a century, diagnosis was dependent on histopathologic examination of post-hysterectomy specimens till the introduction of noninvasive ultrasound and MR techniques [13]. Since then, several studies have illustrated high sensitivities and specificities for both two-dimensional transvaginal sonography (TVS) and magnetic resonance imaging (MRI) [13–17]. Current treatment options for symptomatic adenomyosis include hysterectomy, medication, conservative surgery, or minimally invasive techniques including uterine artery embolization [18]. To date, hysterectomy remains the definitive treatment. This is mainly due to difficult diagnosis, the diffuse nature of the disease, and little evidence-based literature needed to standardize treatments [19]. This consequently results in a management dilemma, particularly in symptomatic patients who wish to preserve their uterus [18].

Uterine artery embolization (UAE) was first described in 1995 by Ravina et al. [20] then later established as an effective treatment option for patients with symptomatic uterine fibroids [21, 22]. Since then, UAE has been investigated as a noninvasive treatment option for adenomyosis with initial promising results [23, 24]. What remains to be known is whether UAE can be validated as a safer, noninvasive, uterine-sparing alternative to hysterectomy. This article summarizes current trends in management of uterine adenomyosis with special focus on the emerging role of UAE.

### Etiology

The precise etiology and pathophysiology leading to the development of adenomyosis remains undetermined. Several theories have been introduced, including traumatic, immunological, hormonal, metaplastic, and stem cell [25]. Traumatic and immunological theories suggest disruption of endometrial-myometrial interface with invagination of eutopic (normally located) endometrial cells [26, 27], while hormonal, metaplastic, and stem cell theories rely on the altered behavior of atopic (displaced) cells [28–30]. These mechanisms, in addition to various risk factors, such as age, parity, previous uterine surgery, smoking, ectopic pregnancy, antidepressant, and tamoxifen therapies, are believed to contribute to the development of adenomyosis [19]. Regardless of etiology, histopathologic features remain the same, and definitive diagnosis is established by the presence of “ectopic, non-neoplastic, endometrial glands and stroma surrounded by hypertrophic and hyperplastic myometrium” on hysterectomy specimens [1].

### Diagnosis

Adenomyosis remains an underdiagnosed condition. This is largely due to lack of pathognomonic symptoms related to this condition [31]. Symptomatic patients varyingly present with menorrhagia, dysmenorrhea, chronic pelvic pain, dyspareunia, and subfertility [32–34], and up to 30% of patients are asymptomatic [34]. Furthermore, confounding coexisting pathologies (usually fibroids and endometriosis) add to the difficulty of diagnosis, as both entities present with similar clinical features [31].

#### Role of ultrasound and MRI in diagnosis

With the introduction and advancement of ultrasound and MR techniques, various criteria have been utilized in the noninvasive narrowing of the clinical differential [15, 35, 36], determining the depth of myometrial invasion and monitoring treatment response [37].

**Fig. 1:**
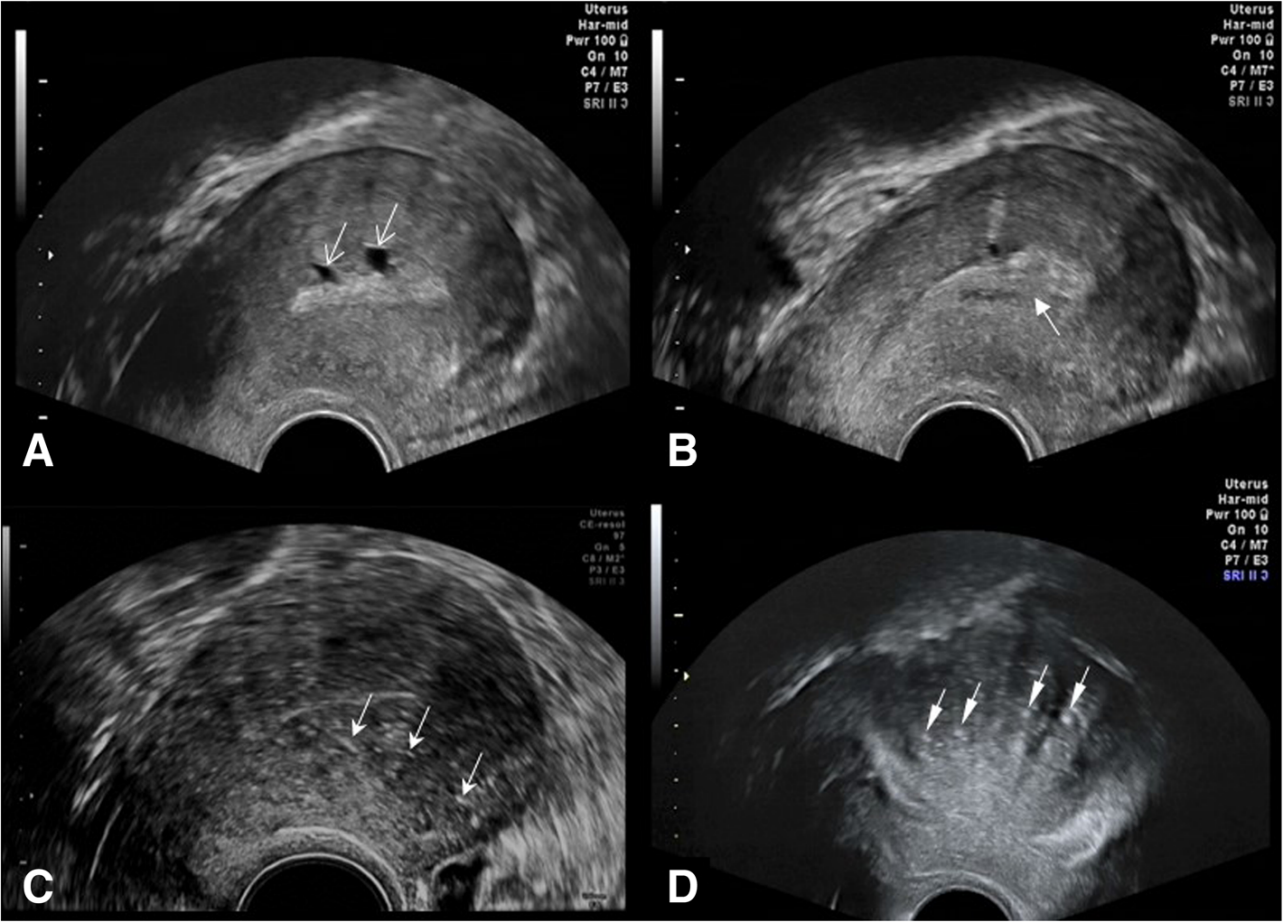
Direct and indirect imaging features of adenomyosis on ultrasound. **a** Small posterior wall myometrial cysts (open arrows). **b** Poorly defined endo-myometrial interface (solid arrow). **c** Diffuse myometrial heterogeneity with hyperechoic linear striations (three arrows). **d** Diffuse asymmetric widening of the posterior myometrial wall with hyperechoic nodules (four arrows)

Transvaginal ultrasound (TVS) represents a cost-effective initial screening modality for adenomyosis. Ultrasound features of adenomyosis can be divided into direct or indirect features (Fig. 1). Direct features are due to the presence of endometrial tissue within the myometrium, and indirect features are due to a hypertrophied myometrium as described by Atri et al. [38]. Table 1 describes ultrasound features of adenomyosis as described in previous literature [14, 16, 38–43]. To report the diagnostic accuracy of TVS in adenomyosis, several meta-analyses have been published [17, 44–46]. Estimated pooled sensitivities of 72 to 82%, pooled specificities of 81 to 85%, and pooled positive likelihood ratios 3.7 to 4.67 have been reported [17, 44]; however, one meta-analysis suggested that variability between studies does not allow for accurate statistical pooling [45]. With the introduction of color and power Doppler ultrasound, three-dimensional TVS and elastography techniques to the work-up of adenomyosis, there is promise for further improvement in diagnostic accuracy [46].

**Table 1:** Direct and indirect imaging features of adenomyosis

Imaging feature	Ultrasound description	MR description
Direct features	Tiny myometrial cysts	Tiny myometrial cysts
	Hyperechoic nodules or striations	Myometrial foci of high signal intensity on T1-weighted images
	Poor definition of the endometrial-myometrial interface	
Indirect features	Diffuse myometrial heterogeneity associated thin hypoechoic linear striations within a heterogeneous myometrium	Junctional zone thickening
		Abnormal myometrial signal intensity
	Diffuse asymmetric or symmetric widening of the myometrial walls	Large, regular, asymmetric uterus without leiomyomas

**Fig. 2:**
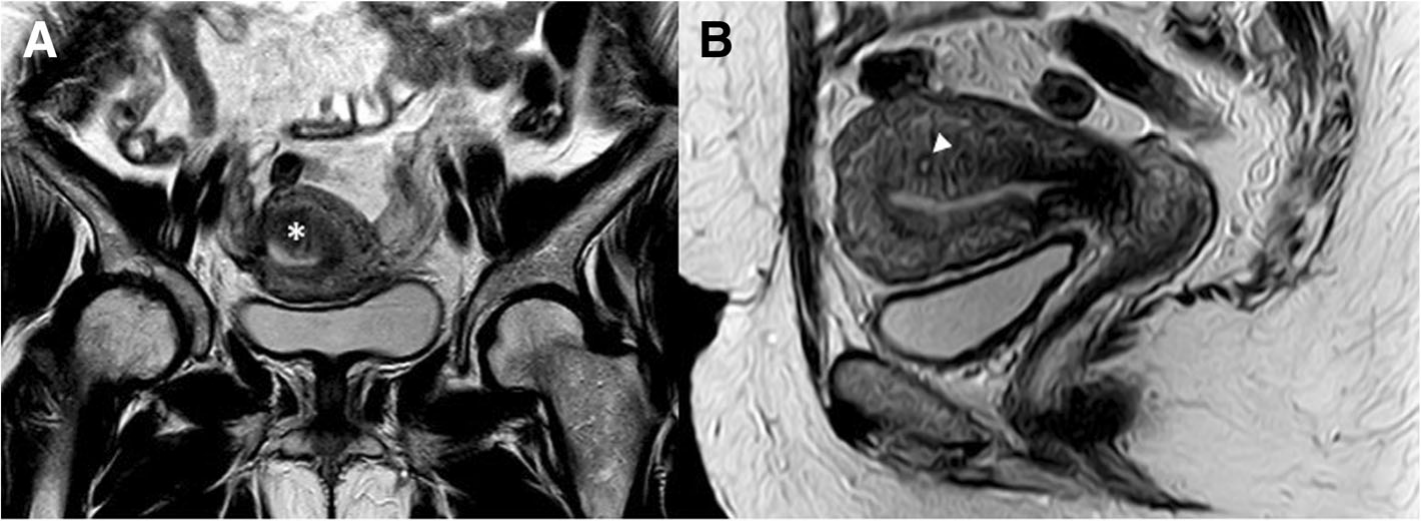
Coronal (**a**) and sagittal (**b**) T2W 1.5-T pelvic MRI images of a 42-year-old female with persistent pelvic pain following cesarean section show focal thickening of the posterior uterine wall transitional zone (asterisk) with tiny myometrial cyst (solid arrow head), suggesting focal adenomyosis

Magnetic resonance imaging (MRI) represents a second line, detailed imaging modality for the detection of adenomyosis (Fig. 2). Similar to ultrasound, various direct and indirect features can be used to describe adenomyosis, but need more knowledge of uterine anatomy and its cyclic variations [36]. Table 1 describes MRI features of adenomyosis as described in previous literature [14–16, 35, 47]. Few prospective studies have evaluated the diagnostic accuracy of MRI in the diagnosis of adenomyosis [15, 16, 48]. These studies have reported sensitivity between 70 and 93% and specificities between 86 and 93%. Despite being less operator dependent, MRI needs more reader experience and optimization of imaging technique to achieve higher diagnostic accuracy [36].

### Classification of adenomyosis

The use of complex imaging techniques has revealed various subtypes of adenomyosis, often associated with histopathologic variation in glandular and muscular components [31]. Furthermore, initial studies have linked various imaging criteria to symptoms of adenomyosis [49–51]. Therefore, the need for a more holistic approach to identify various disease characteristics incorporating symptomatology, morphology, and pathologic features is rising in order to improve the diagnostic accuracy and adequately guide treatment decisions. Important factors to be included in classification systems would be the site and location of pathology, configuration, and size/volume relative to the total myometrial thickness [31]. Most recent ultrasound and MR classification and reporting systems have been developed by Van den Bosch et al. [52] and Bazot [36, 53] respectively, but the clinical relevance remains to be tested.

### Treatment

As with many aspects of adenomyosis, treatment remains controversial. Important factors to be considered and discussed with patients are age, symptom severity, desire for future conception, and associated comorbidities [32, 54, 55]. Recent studies estimate a prevalence of adenomyosis among females younger than 40 years to be about 20–30%, while in the rest of the patients, diagnosis is usually established in the fourth or fifth decade [55–57]. Furthermore, diffuse adenomyosis, depth of invasion, and coexisting fibroids and/or endometriosis are associated with increased frequency/severity of symptoms and reproductive complications [31, 49, 58–60]. Currently, treatment is on a case by case basis, and hysterectomy remains the definitive treatment in patients who are willing and do not wish for future fertility. With the introduction of assisted reproductive techniques, delayed age of conception, and availability of minimally invasive treatment options, the shift from hysterectomy as the “go to” option seems inevitable.

#### Medical treatment

Medical treatment is the first-line treatment option for adenomyosis aiming to relieve symptoms and maintain fertility with the least possible side effect. This is achieved by disrupting pathways leading to inflammation, neuroangiogenesis, and impaired apoptosis [61]. Currently, several hormonal and non-hormonal options, namely gonadotropin-releasing hormone (GnRH) analogues, progestins, combined oral contraceptives, and non-steroidal anti-inflammatory drugs are being used in an “off label” manner for the symptomatic treatment of adenomyosis [57, 62]. Also, newer drugs, such as aromatase inhibitors, have been investigated by Badawy et al. and Tosti et al. [63, 64], while other therapies such as selective progesterone receptor modulators, GnRH antagonists, valproic acid, and anti-platelet therapies are still under investigation [55].

The main advantage of medication is symptomatic relief without the need for surgical treatment. Nevertheless, many drawbacks still need to be addressed. This includes the temporary relieve of symptoms, and the common (i.e., menopausal symptoms, irregular bleeding, amenorrhea) and occasionally severe (i.e., thromboembolic) side effects of some drugs. Lack of evidence needed to base choice of drugs also raises the need to perform research into the comparative efficacy of currently used drugs and develop a more standardized approach for patients wanting to conceive while using medication. With a better understanding of pathogenetic mechanisms of adenomyosis, advances in drug development will soon be possible [55].

#### Minimally invasive techniques

These are second-line treatment options aiming to cure symptoms and preserve the uterus in patients with failed medical therapy. Conservative surgical treatments aim to remove adenomyosis and preserve the remaining normal uterine muscles through laparotomy, laparoscopy, hysteroscopy, or combined approach. Excisional adenomyomectomy involves the complete removal of focal lesions (adenomyomas), while myometrectomy is the surgical debulking of diffuse adenomyosis. Non-excisional treatments aim to induce necrosis of focal or diffuse adenomyosis through selective vascular occlusion or focused ultrasound/thermal energy without direct tissue dissection. In some cases, a combination of surgical and non-excisional methods, i.e., hysteroscopic resection/ablation, are used to achieve maximum cytoreduction and reduce myometrial tissue damage.

#### Conservative surgical treatment

Debulking/cytoreductive surgeries aim to remove visibly diseased tissue with repair of the remaining myometrial tissue [65]. Several laparotomic techniques have been described, including wedge resection and its modifications, transverse H-shaped incision [66], wedge-shaped uterine wall removal [67], double and triple flap [68, 69], and asymmetric dissection methods [70]. Laparoscopic techniques have also been described in more focal pathology, where longitudinal or transverse incisions [71, 72] are used to access adenomyotic lesions followed by resection using monopolar needle or laser knife [73, 74], bag removal, and repair in layers or using double flaps [72, 75]. To date, there is no consensus on the best surgical method, but initial results are promising. In a systematic review by Grigoris et al., dysmenorrhea reduction, menorrhagia control, and pregnancy success rates ranged from 81 to 82%, 50 to 69%, and 47 to 61% among partial versus complete adenomyosis excisions respectively [76], and a recent review by Younes et al. showed 75% symptom relief on short-term follow-ups [77]. The main issue with conservative surgical methods is the high risk for complications, i.e., uterine rupture and complicated pregnancy [54, 65] (especially in diffuse lesions and on long-term follow-up), making this option safer in focal adenomyomas.

#### Hysteroscopic resection/ablation

Hysteroscopic resection/ablation is a combined treatment method involving the dissection and or coagulation of cystic adenomyotic lesions and crypts [78–82]. Hysteroscopic resections can be performed using yttrium aluminum garnet (YAG) laser, rollerball resection, thermal balloon ablation, cryoablation, circulated hot fluid ablation, microwave ablation, bipolar radiofrequency ablation, and electrocoagulation [19].

#### High-intensity focused ultrasound (HIFU)

High-intensity focused ultrasound (HIFU) is the use of intense ultrasound energy directly targeting abnormal tissues and their vascularity through heating and cavitation, sparing the normal surrounding tissues. This process can be guided and monitored through MRI or ultrasound [83]. High-intensity focused ultrasound has been used since 2008 for the treatment of adenomyosis [84]. Since then, literature has shown promising results regarding symptom relief and uterine preservation with few reported complications (namely pain, numbness, vaginal or urinary discharge, fever, skin burn, or contact dermatitis) [83]. Recent studies have also investigated the use of ultrasound contrast agents (microbubbles) and hormonal (GnRH) and non-hormonal (metformin) treatments to enhance the efficacy of HIFU. Microbubbles are believed to improve the ablative effects of HIFU by changing the acoustic characteristics, thus increasing energy deposition in target tissues, while GnRH and metformin inhibit cellular proliferation and induce apoptosis [85–87]. Limited literature on treatment outcomes for HIFU in adenomyosis has shown highly variable results regarding symptom and uterine volume reduction [88–97]. Rates of menorrhagia, dysmenorrhea, and uterine volume reduction varied widely from 12.4 to 44.8%, 25 to 100%, and 12.7 to 54% respectively, increasing gradually overtime (from 1 to 24 months). Nevertheless, paucity of literature comparing HIFU to other minimally invasive treatment options, limited availability, overall cost, unknown fertility outcomes, and strict indications, including lesions no more than 10 cm in diameter [88, 90], no pelvic adhesions [84, 89, 90, 93], body weight less than 100 kg [98], and abdominal wall thickness less than 5 cm [93] may limit its widespread use.

#### Uterine artery embolization (UAE)

**Fig. 3:**
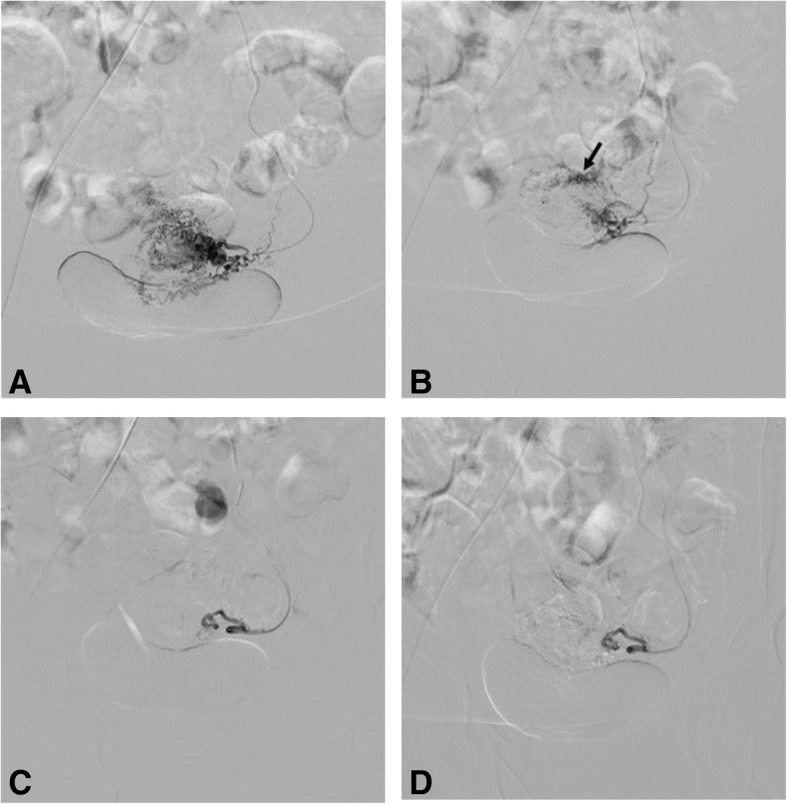
Digital subtraction angiography (DSA) images (of the same patient in Fig. 2) with selective injections of the left (**a**) uterine artery demonstrate with multiple tortuous uterine artery branches and (**b**) lesion blush (most prominent at the anatomic site of the posterior uterine wall). Right uterine artery injection (not shown) was unremarkable for pathology. Post-embolization DSA images show occlusion of toursous feeding vessels (**c**) with absence of lesion blush (**d**)

Uterine artery embolization is the use of transarterial catheters aiming to induce more than 34% necrosis within adenomyotic tissues [99, 100]. The technique for UAE in adenomyosis is similar to that used in fibroids. In many parts of the world, UAE is performed under conscious sedation. Vascular access is gained through a femoral or radial artery puncture using 4–6-French (F) arterial sheath for femoral [99, 101] and 4-F sheath for radial access [102]. Under fluoroscopic guidance, aortography is followed by selective and super selective arteriography using 4–5-F catheters for the internal iliac and 2–3-F microcatheters for the uterine artery and its branches respectively. Embolization is usually performed using variable-sized permanent particulate agents [103, 104]. Special attention is paid to visualization of the cervicovaginal and ovarian artery branches (Fig. 3). Distal embolization avoids vaginal necrosis and unwanted reflux of microspheres into the ovarian artery [105].

Despite being established in fibroids as a cost-effective, short recovery alternative to surgery with minimal complications [19, 23, 100], it was believed to have lower efficacy in adenomyosis [106]. In the past 15 years, UAE has been considerably studied for the treatment of symptomatic adenomyosis [107]. Earlier studies by Popovic, Keung, and Zhou et al. demonstrate long-term improvement in patient symptoms (in over 60% of patients) and a short-term decrease in uterine volumes (in over 20% of patients), especially in vascular lesions [23, 107, 108]. Current literature by Dueholm and Bruijn et al. show up to 67% long-term (40 month) treatment success and up to and 72% patient satisfaction rates respectively [24, 100]. In the latest systematic review and meta-analysis by de Bruijn et al., patients were divided into four groups to report short- and long-term outcomes. Short-term improvement was achieved in 89.6% of patients with pure adenomyosis and 94.3% of patients with adenomyosis with fibroids, while long-term improvement was achieved in 74.0% of patients with pure adenomyosis and 84.5% of patients with adenomyosis with fibroids [109].

Overall, UAE shows favorable clinical outcomes, but randomized controlled trials are still lacking [110]. In an attempt to fill this gap in knowledge, the “Quality of Life after Embolization vs Hysterectomy in Adenomyosis” (QUESTA) trial was set up. This multicenter non-blinded randomized controlled trial is currently ongoing in the Netherlands. It has started since November 2015, and its primary outcomes are expected by May 2020 [101]. The calculated sample size for this trial was 96 patients (divided into 52 embolization and 34 hysterectomy, including a 10% expected drop-out) made on assumptions from the embolization versus hysterectomy (EMMY) trial outcomes [111].

Inclusion criteria were premenopausal women with symptomatic pure adenomyosis or dominant adenomyosis when both adenomyosis and fibroids coexist and women with an indication for hysterectomy (either failed or refused medical treatment). Exclusion criteria were patients under 18 years of age, pelvic infection, suspected or confirmed malignancy, current or future desire to conceive, any absolute contraindication to angiography, deep infiltrating endometriosis requiring surgery or obstructing the bowel, or coexisting hysteroscopically removable submucous fibroids. Following selection, TVUS and MRIs were performed to confirm the adenomyosis and eligible patients are informed of the trial. Patients with written informed consents were randomly allocated (in a 2:1 ratio) between both experimental intervention (UAE) and standard care control groups (hysterectomy), while patients refusing randomization are given the standard of care (hysterectomy) [101].

Following the procedure (UAE or hysterectomy), patients are followed up immediately, then at 6 weeks, 3 months, 6 months, 12 months, and 24 months using an online questionnaire system. Three outcome parameters were measured. *Primary outcomes* (quality of life) were measured at 6, 12, and 24 months using a combination of World Health Organization Quality of Life Scale and Short Form-12 Questionnaires. *Secondary outcomes* (clinical, symptom and quality of life, recovery related, cost utility analysis, laboratory, and pathology outcomes) were measured at 6 weeks and 3, 6, 12, and 24 months. *Imaging outcomes* were also determined to identify potential predictive parameters for therapy effect using specific TVUS criteria (uterine size/fibroid volume reduction in case of associated fibroids, vascular index by 3D power Doppler) at baseline, 6 weeks, and 6 months and MRI criteria (uterine size/fibroid volume reduction in case of associated fibroids, junctional zone reduction, infarction rate, and presence of endometriosis) at baseline and at 6 months postprocedure [101].

### UAE as an alternative to hysterectomy

To date, UAE seems to be the most investigated and highest potential minimally invasive treatment option for adenomyosis. Results of ongoing randomized controlled (QUESTA) trial will soon show whether UAE can be validated as a treatment option for adenomyosis. Although comparative information regarding quality of life, patient satisfaction, side effects, and complications post UAE versus hysterectomy will soon be available, questions regarding fertility post UAE remain to be answered. Current American College of Obstetrics and Gynecology and Society of Interventional Radiology guidelines still consider desire for future fertility a relative contraindication to UAE, but conflicting reports regarding effects of UAE on fertility [112] still give room for debate. Nevertheless, further randomized studies are still needed to give a clear answer for physicians and patients alike.

In conclusion, lack of information is the main hurdle to overcome the complexity in management of adenomyosis. With randomized controlled trials and more evidence-based research, optimal treatment protocols can be developed according to patient needs. Whether or not UAE can replace hysterectomy will largely depend on the results of ongoing QUESTA trial and other randomized trials comparing fertility outcomes among minimally invasive therapies.

## Abbreviations

EMMY: Embolization versus hysterectomy
GnRH: Gonadotropin-releasing hormone
HIFU: High-intensity focused ultrasound
QUESTA: Quality of Life after Embolization vs Hysterectomy in Adenomyosis
UAE: Uterine artery embolization
YAG: Yttrium aluminum garnet
